# Human Systems Biology and Metabolic Modelling: A Review—From Disease Metabolism to Precision Medicine

**DOI:** 10.1155/2019/8304260

**Published:** 2019-06-09

**Authors:** Claudio Angione

**Affiliations:** ^1^Department of Computer Science and Information Systems, Teesside University, UK; ^2^Healthcare Innovation Centre, Teesside University, UK

## Abstract

In cell and molecular biology, metabolism is the only system that can be fully simulated at genome scale. Metabolic systems biology offers powerful abstraction tools to simulate all known metabolic reactions in a cell, therefore providing a snapshot that is close to its observable phenotype. In this review, we cover the 15 years of human metabolic modelling. We show that, although the past five years have not experienced large improvements in the size of the gene and metabolite sets in human metabolic models, their accuracy is rapidly increasing. We also describe how condition-, tissue-, and patient-specific metabolic models shed light on cell-specific changes occurring in the metabolic network, therefore predicting biomarkers of disease metabolism. We finally discuss current challenges and future promising directions for this research field, including machine/deep learning and precision medicine. In the omics era, profiling patients and biological processes from a multiomic point of view is becoming more common and less expensive. Starting from multiomic data collected from patients and N-of-1 trials where individual patients constitute different case studies, methods for model-building and data integration are being used to generate patient-specific models. Coupled with state-of-the-art machine learning methods, this will allow characterizing each patient's disease phenotype and delivering precision medicine solutions, therefore leading to preventative medicine, reduced treatment, and* in silico* clinical trials.

## 1. Introduction

With the advent of bioinformatics and computational biology, computational and mathematical techniques can provide accurate simulation of biological processes. The most widely used approaches to analyze omics data mainly focus on genomics, transcriptomics, and proteomics, through differential expression or network-based coexpression analysis. However, genes and their expression alone do not always constitute a reliable indicator of cellular phenotype. When characterizing a phenotypic outcome, relying solely on gene or protein expression profiles will miss the highly nonlinear interaction between these biological layers. Such approaches often overlook the metabolic level, the dense network of biochemical reactions occurring in a cell with the aim of converting nutrients into energy and cellular building blocks.

Being the best-characterized network in biological systems and also the closest to the phenotype, metabolism is arguably the best indicator for the cell physiological state [1]. Once considered only a passive result of the state of a cell, it is now widely recognized as a main contributor to cellular behavior. More specifically, it is a key player in a number of diseases, including diabetes, neurodegenerative diseases, and cancer, where altered metabolism is now accepted as a hallmark [2].

**Figure 1:**
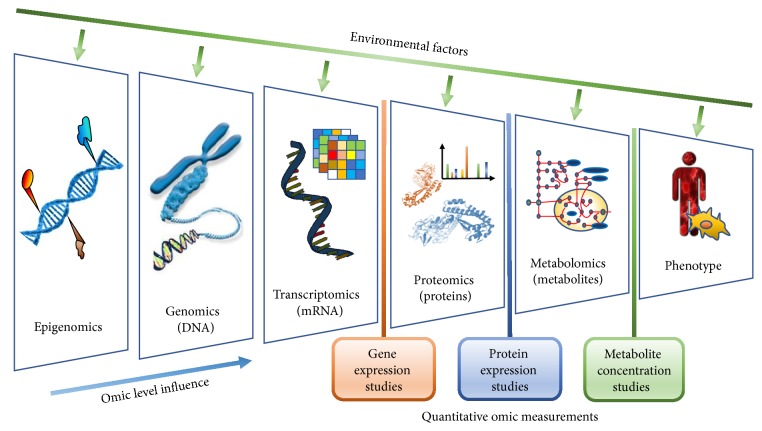
*The observable phenotype of a cell is a result of complex interactions and feedback loops among several omic layers, each influenced by environmental perturbations.* In order of “distance” from the cell phenotype, these are epigenomics (epigenetic markers that affect gene activity and expression), genomics (DNA containing the genetic code of the cell), transcriptomics (the RNA encoded by the genome), proteomics (the set of proteins produced as a result of gene expression and subsequent posttranslational modifications), and metabolomics (the set of metabolites and metabolic reactions taking place in the cell). Although each omic layer can be studied alone, no single-omic layer has achieved a satisfactory correlation with the phenotypic observables. As a result, in recent years, a multiomic approach has been adopted where all layers are considered together, and the effect of interactions and feedback is taken into consideration.

The recent availability of high-throughput data regarding multiple layers of biological organization (omics) allows mapping cellular processes at the levels of genes, mRNA, proteins, and metabolites (Figure 1). In a single experiment, these measurements are often at both the “genotype” level (i.e., referring to the genetic elements on a genome) and at the “phenotype” level (the form and function of the cell). A fundamental question in systems biology is the definition and understanding of the genotype-phenotype relationship [3]. A mechanistic link between genotype and phenotype is offered by genome-scale metabolic models, which contain all known biochemical reactions occurring in a cell. Such models have been generated taking into account decades of studies in biochemistry and in most cases are able to predict the cellular phenotype with high accuracy.

Constraint-based modelling is the most widely used approach to model the behavior of metabolism, often assuming that cells have to fulfill a given task (e.g., ATP production, growth, or proliferation) or to optimize the production of a given compound. Such models have two main advantages: first, they do not need dynamic or kinetic data as they are based on mass balance across the metabolic network; second, they are suitable for integration of different omic layers at genome scale to improve their predictive performance. In particular, multiomic “vertical” integration methods have been proposed to include omic layers (mainly transcriptomics and proteomics) [4–6]. Conversely, “horizontal” integration methods have focused on modelling different environments, cancers or growth conditions starting from the same model [7–11]. Such multiomic integration in genome-scale models has provided a mechanistic link between the genotype and their phenotypic observables [12–15]. This is a key added feature that such models possess if compared with genome-wide association studies (GWAS), which are able to associate gene variations to phenotypic traits, but not to provide a mechanistic explanation of the associations observed [16].

In this review, we focus on metabolic systems biology, and in particular on human metabolic modelling. Originally tested and validated with microorganisms, metabolic systems biology is now becoming widespread for human tissues and biomedical applications [17]. We provide a comprehensive review of the metabolic models developed so far for human cells, discussing their strengths and limitations. We show that the main focus of the metabolic modelling community has shifted from increasing the size of the models—a “size-focused modelathon approach”, often at the expense of reliability of predictions—to team-based curation approaches where gap filling algorithms and extensive manual curation improve the reliability of the predictions without necessarily increasing (and in fact often decreasing) the number of components in the model. We also outline key success stories, current challenges, and future directions for the human metabolic modelling field, including the integration with machine learning methods and precision medicine.

## 2. Metabolic Systems Biology

Understanding the role of individual components in a biological system is an important step to elucidate or predict its behavior. However, a major theme in systems biology is investigating these systems with an integrated approach. In fact, studying the interactions between different components also gives insights into the functioning and behavior of the components taken independently [18].

Metabolism is the biggest biological network fully described. It is a surrogate of the phenotype, being able to elucidate phenotypic changes caused by single or combined omics or physiological factors. It also has the advantage of being the only system that can be entirely modelled at genome-scale. As a result, it plays a central role in elucidating the genotype-phenotype relation. Systems-based approaches have been successfully applied over the last decade to investigate metabolic networks, composed of a set of chemical reactions and a pool of metabolites. The first examples of metabolic systems biology appeared in 1999 and were focused on modelling, connecting, and simulating several cellular processes [19]. Whole-cell modelling, named the “grand challenge of the 21st century”, is still an active area of research [20–24]. In metabolic systems biology, the value of modelling cell metabolism is not merely explanatory of a biological process, but also predictive. A model can be used to suggest hypotheses that can be tested or to pinpoint unexpected behaviors that can be further investigated* in vitro*.

There are different methods to model a metabolic system: steady-state analysis (e.g., FBA) involves a set of linear equations, while kinetic simulations involve ordinary differential equations (ODEs). Each variable represents the variation of a metabolite concentration, in a dynamic or steady state, where the concentration depends on the rates of the reactions that produce and consume that metabolite [25]. Kinetic models do not assume steady state and therefore are able to model highly dynamic mechanisms, including allosteric and posttranslational regulation, metabolite concentrations, and thermodynamics. Such ODE-based systems contain a large number of equations (differential or algebraic) and require unique kinetic parameter values. They are highly effective at predicting the behavior of small systems where sufficient experimental data can be collected for model calibration and parameter estimation [26]. Furthermore, unlike standard FBA-based methods, reaction kinetics can be accounted for, and metabolite concentrations can be modelled explicitly, and therefore intracellular metabolomics data can be integrated directly [27].

For large systems, however, the use of kinetic modelling remains challenging. The increasing demand for systems-level genome-scale analyses has recently led to the widespread use of constraint-based steady-state models and their unsteady-state extensions. This has also been facilitated by the increasing availability of multiomics data and phenotypic information, used to constrain the model dynamically and therefore often compensating for the lack of regulatory and kinetic modelling.

Following a number of successful attempts at building multiscale kinetic and constraint-based models [28, 29], achieving the right combination (and therefore trade-off) of kinetic modelling and steady-state assumptions is likely the next step for the metabolic modelling community. Techniques like unsteady-state FBA (uFBA) [4] have been recently developed to relax the steady-state assumption in genome-scale models, with the goal of modelling dynamic cellular states derived from changes in the concentration of internal metabolites. An approach to integrate reaction kinetics with steady-state metabolic networks has also been proposed, where the genome-scale information of shared metabolites among different reactions is used to inform the interactions between the reactions and predict metabolite concentrations in a network kinetics approach [30]. More generally, due to the larger range of predictions that kinetic models can perform compared to steady-state approaches, expanding them towards the genome scale or using information derived from genome-scale model simulations is a promising direction that will increase their spatial and temporal resolution.

### 2.1. Genome-Scale Metabolic Models

Genome-scale metabolic models contain all known metabolic reactions in an organism and can therefore serve as functional databases of cell-specific metabolism. A genome-scale model is built using the following process. First, a draft reconstruction is generated starting from the genome and including all the genome-encoded metabolic reactions. The draft reconstruction also includes annotated enzyme, reaction, and pathway data from databases like KEGG [31], BioCyc [32], and BRENDA [33]. Details on which genes control each reaction are also included. Then, a sequence of manual curation steps improves the draft reconstruction, by gathering evidence to prove or disprove the presence of a reaction in the network of the organism.

The construction of genome-scale model represents the starting point for flux balance analysis (FBA, see following subsections). Finally, the model is run and validated by comparing its predictions with existing experimental results, and new* in silico* experiments are performed to further improve and validate the model. For more details on how to build a metabolic model from the DNA sequence of an organism, the reader is referred to full protocols [34–36].

**Figure 2:**
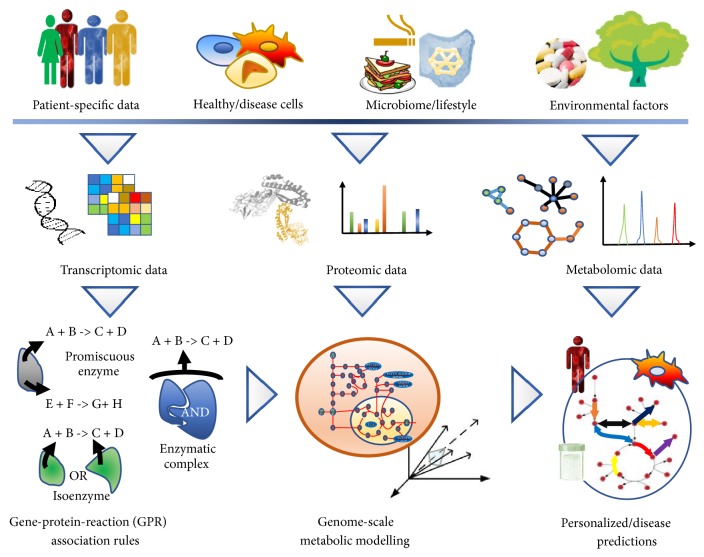
*The integration of different types of omics data can be used to infer tissue- and condition-specific intracellular metabolic flux distributions.* Intracellular metabolic reactions provide the cell with basic biochemical building blocks, as well as energy and a thermodynamically favorable environment to sustain its life. Patient-specific data, molecular information, lifestyle, and environmental factors affect different omic levels. As a consequence, transcriptomic, proteomic, and metabolomic data need to be integrated to determine gene-protein association rules and to build genome-scale models used for personalized predictions. Given the large effect of environmental factors on omics level, determination of system-level changes in intracellular metabolic fluxes is important for understanding the fundamental mechanisms of metabolic responses to perturbations. Indeed, environmental factors affect omics data on different levels, form epigenomics to the cell phenotype. Omic-augmented genome-scale metabolic reconstructions have proved successful due to the ability to integrate omic measurements at genome scale and to give mechanistic insights into the genotype-phenotype relationship.

Most genome-scale models are annotated with curated gene-protein-reaction associations (GPR rules), linking genes with enzymes (Figure 2). Such annotations pave the way for overlaying multiomic data on the models, using them as omic-scaffolds (see Section 2.3). Since omic data can be quantified numerically and in a condition-, tissue-, and patient-specific way (e.g., transcriptomic profiles, protein levels, and metabolite concentrations), models with GPR rules can serve as a baseline for generating personalized metabolic models. For instance, personalized predictions using such models can lead to precise phenotypic characterization of patients (see Section 4).

### 2.2. Constraint-Based Modelling and Flux Balance Analysis

Flux balance analysis (FBA) is the most widely used constraint-based technique to predict flux distributions and network capabilities in genome-scale models [37]. FBA has proved useful thanks to its ability to handle large networks and predict genome-scale flux distributions. It requires information about biochemical reactions and stoichiometric coefficients but does not involve kinetic parameters. This makes it well suited to metabolic engineering studies that identify and characterize optimal perturbations such as different substrates or genetic interventions (e.g., knockouts) leading to obligatory coupling between the growth rate and the overproduction of the desired metabolite [38–42]. In general, FBA is a powerful tool for predictions of cell behavior under different metabolic conditions.

FBA is a linear programming technique that models the steady-state condition in a chemical reaction network [43]. The FBA representation of a genome-scale model is built based on a stoichiometric matrix *S*, containing the stoichiometric coefficients of each metabolite (on the rows) in each reaction of the network (on the columns). A stoichiometric coefficient of a metabolite in a given reaction is positive if the metabolite is produced by the reaction and negative if consumed. An underdetermined linear system of equations *Sv* = 0 is defined from the stoichiometric matrix, where the unknowns are represented by the vector *v* of reaction flux rates. Additional constraints are included as lower and upper bounds of the fluxes in *v* (*v*^min^ ≤ *v* ≤ *v*^max^). These account for the growth or physiological condition, and can be used to incorporate omics data. One or more cellular objectives (usually growth- or energy-related, e.g., biomass or ATP), or a linear combination thereof, are finally selected to be maximized or minimized under the above-mentioned constraints, therefore solving a linear program.

FBA is therefore based on two main assumptions:

- Homeostatic assumption: the organism has reached a steady state where the metabolite concentrations are constant and a set of nutrients are being constantly converted to generate biomass.
- (Multilevel) optimality: in each state, the organism tends to maximize one or multiple objectives, usually related to growth, biotechnologically relevant compound production (e.g., acetate exchange) and important energy-carrying molecules (e.g., ATP).

FBA is widely used in systems biology to quantify the entire metabolic steady state of a cell and calculate its flux distribution. All known metabolic reactions in a given cell are considered, and they are mathematically described in a way that allows simulation of various states and configurations of the chemical reaction network. Intuitively, the steady-state constraints used in FBA can be thought of as Kirchoff's laws applied to any node representing a metabolite in the network: the flux through each metabolite in the network must be constant, namely the input flux must equal the output flux. The combination of steady-state constraints and capacity constraints on reaction fluxes is a system of linear homogeneous equations and inequalities; thus, its solution space is a convex polyhedral cone representing the feasible flux distributions.

The assumptions of FBA and the reduction of the problem to a linear program can cause some limitations. First, incorporating or predicting metabolite concentrations is challenging, and requires a dynamic FBA [44], relaxing the steady state [4], flux-sum methods [45], or thermodynamic approaches [46]. Second, the reliability of the flux distributions is highly dependent on the objective function chosen (see Section 2.4), on the quality of the reconstruction and on the method used to obtain the solution. Regularized FBA methods help alleviate this issue, as discussed later in this section and in Section 5. Finally, FBA lacks the ability to directly model regulatory effects or posttranscriptional regulation of expression levels. Likewise, changes occurring over quick transients (e.g., perturbations to the cell microenvironment) cannot be modelled dynamically, but can be approximated through step-wise before/after simulations [47]. Although the steady-state rule has been challenged and probabilistic approaches have been proposed to relax the steady-state equality [48], this assumption enables the use of linear systems and linear programming, lowering the computational requirements and enabling fast simulation of genome-scale models in a variety of growth or physiological conditions.

When solving linear programs for FBA, different solvers can give different solutions due to numerical implementation differences and to the existence of multiple alternate optimal solutions [49–51]. While the value of the objective function is the same in all the optimal solutions, the other flux rates can vary. Having a unique solution is therefore important when the full flux distribution is used for further analysis or as a feature of predictive algorithms. To avoid the degenerate solutions provided by standard FBA approaches, parsimonious FBA (pFBA) has been proposed with the aim of minimizing the overall flux carried by the metabolic network after maximization of the main objective [52]. However, in some cases, pFBA has produced less plausible results in central carbon metabolism and in the glycolytic pathway compared to standard FBA methods [53]. Furthermore, pFBA makes use of the L1-norm (minimization of the sum of absolute flux values) which does not guarantee a unique solution as it is not strictly convex [54]. For solving such minimization problems with a guarantee of a unique solution, the L2-norm should be used instead (minimization of the sum of squared flux values). Additional approaches to alleviate the problem of alternate optimal solutions include geometric FBA [55] and sampling [56].

Several tools are available for metabolic model reconstruction, constraint-based modelling, FBA and related analyses. These include COBRA [57], its Python version COBRApy [58], RAVEN [59], PathwayTools [60], and FAME [61]. For a complete list, the reader is referred to the related reviews and comparison papers [62, 63].

### 2.3. Multiomic Flux Balance Analysis

Although some biological outcomes can be elucidated through single-omic analysis (e.g., protein-protein interaction or gene networks), the vast majority of phenotypic observables are a result of more comprehensive interconnections among multiple omics [64]. Regulatory mechanisms also take place at different omic levels (including transcription, translation and metabolic reactions). The complex interplay between these levels is responsible for cell behavior. Multiomic analyses have been proposed to integrate single-omic networks, based on coexpression or interaction networks [65]. However, such methods often omit mechanistic links between omic layers, derived from previous molecular knowledge.

The idea of multiomic FBA is that by mapping omic data onto* in silico* models of metabolism, it is possible to obtain a metabolism-enriched view of any given omic profile. In this context, as well as permitting the simulation of large, usually genome-scale, systems in a few seconds of CPU time, FBA also has the advantage of facilitating the introduction of additional omic layers of experimental data that can be overlaid onto the model, using GPR rules or metabolite annotations to place constraints on the flux bounds. Omic-informed metabolic models consider the mechanistic relation between omics, therefore correctly representing the prior information available on biochemical networks [66]. Multiomic FBA allows assigning the importance of a gene or enzyme through its predicted function, therefore avoiding approximations or statistical methods merely based on the value of its expression level.

Following this approach, FBA and its multiomic modifications have been used to predict the metabolic response to a given condition in light of the multiple cellular objectives that a cell is required to meet [67, 68]. Recent studies have been also aimed at improving the predicting capability of a metabolic model and elucidating the genotype-phenotype relationship through computational analyses across multiple omic levels. Predictions of flux distributions after multiomic data integration, combined with experimental methods, have been successfully used to formulate novel biological hypotheses. These techniques reduce the problem of determining the flux distribution through all reactions in the system, under a given growth or physiological condition, to a tractable linear program, under the assumptions of steady state and optimality. Due to their scalability and precision, these methods have been used widely, e.g., to predict bacterial growth phenotypes in specific environmental conditions [69, 70], to identify novel therapeutic targets against infections [71, 72], to characterize cancer metabolism of different cell lines [73–76], and to generate cancer-vs-normal tissue-specific models for 17 tissues [77]. Further examples of how this approach has been used to characterize disease metabolism are provided in Section 4. For a comprehensive review on multiomic integration techniques in FBA models, the reader is referred to the recent reviews on omic-informed metabolic modelling [78–81].

Ongoing modelling efforts are aimed at incorporating further biological knowledge in the models. To account for enzyme promiscuity when overexpressing or underexpressing a gene, GPR rules have been proposed as additional rows of the stoichiometric matrix, as an enzyme-by-reactions submatrix [53]. For instance, if multiple reactions are catalyzed by the same promiscuous enzyme, the change in flux as a result of underexpression/overexpression of the enzyme is distributed among the reactions (also considering the other enzymes participating in the reactions), rather than triggering* a priori* an equivalent flux increase/decrease in all the reactions. Codon usage can also be integrated through the GPR rules [71]. Splice isoform annotations, initially lost and then simply ignored in human reconstructions, have also received recent attention [82, 83].

Soft constraints can be used to integrate omics data in a reaction-specific fashion, rather than with a single mathematical rule modifying the constraints for all the reactions [84]. Further constraints have been also added to integrate transcriptional regulatory models and metabolic models. The idea is that a constraint models the correlation between the expression level of a target gene and that of its regulating genes [6]. To further analyze the reaction flux distributions predicted by multiomic FBA, sensitivity analysis can be carried out on genes, reactions or pathways. Definitions of sensitivity scores have been based on effects of gene knockouts [85], real-valued gene perturbations [69], or the role of each metabolite in reducing thermodynamic uncertainty in the model [86].

### 2.4. Choosing an Objective Function and Accounting for Multiple Cellular Goals

The selection of an appropriate objective function is still a challenge in metabolic modelling. A common assumption in systems biology is that cells tend to optimize their metabolic network in order to maximize the growth rate (biomass). This is however still a matter of debate [87], both in terms of its composition [88] and because in many cases the best objective for a cell is not growth-related [89]. While models of microorganisms can assume that the cell aims at maximizing growth rate, human cells might not necessarily aim for maximum biomass (although this is widely accepted as an objective for cancer cells). Furthermore, it is now evident that the cellular goal can change between different cells in a tissue, between tissues, and also over time for the same cell. For tissue-specific studies, starting from a generic human metabolic reconstruction (e.g. Recon) and using the general-purpose biomass composition of generic human models can prove unreliable. Recent studies are therefore moving towards a cell-specific estimation of the biomass compositions [13, 90]. Algorithms for generating and standardizing the biomass reaction [91, 92], and for automatically generating an objective function have also been proposed [93].

Especially in the context of human metabolism, the question* ‘what does a particular cell do?'* has often more than one correct answer. There is increasing evidence that cells have to cope with multiple, usually competing, objectives to optimize simultaneously [94]. A single FBA objective function is not able to capture all of them. It is also likely that metabolism is not fully optimized for any particular objective [95], and evolution has shaped cells in order to reach an optimal trade-off between all objectives [96].

Optimization processes have therefore been proposed that take into account multiple objectives (e.g., protein or energy production, detoxification, proliferation) with the advantage of ensuring metabolic flexibility for possible reorganizations performed during adaptations to changes in the environmental conditions [97]. These methods include a probabilistic approach [98], lexicographic ordering [99], and the reduction to a single objective through the definition of weights for the objectives [100, 101]. Approaches based on these ideas are fast and can generate a Pareto front; however, they miss suboptimal solutions, solutions in nonconvex regions [102, 103], or they give preference to one objective [104], therefore addressing a multilevel problem rather than a strictly multiobjective problem. Although slower, methods based on evolutionary algorithms are able to explore nonconvex trade-offs without requiring the combination of the objectives into a single objective function [105–107]. Exploring a set of trade-off solutions between competing objectives, rather than a single biomass-maximizing solution, also accounts for suboptimal solutions [108].

## 3. 15 Years of Human Metabolic Modelling

The study of human metabolism is becoming increasingly important for biomedical applications as an approach for understanding many diseases and aspects of health [109, 110]. A systems-level understanding of metabolic behavior is enabled by the availability of high-quality genome-scale reconstructions integrating extensive metabolic information from various resources. The first genome-scale metabolic reconstruction efforts focused on bacteria (*Haemophilus influenzae* [111], followed by* Escherichia coli* [112]), due to their simplicity, the available genome sequences, and the well-known mechanisms of substrate utilization. Reconstructions of human metabolism, which required a much larger number of pathways and a larger pool of essential nutrients, were attempted at a later stage, after the publication of the draft and complete human genome sequences in 2001 and 2004 [113, 114].

HumanCyc [115] (in 2004) and Reactome knowledgebase [116, 117] (in 2005) were the first successful attempts to build a curated collection of biochemical reactions in human cells. The first models of human metabolism published in 2007—Recon 1 [118] and EHMN (Edinburgh human metabolic network reconstruction, and its subsequent compartmentalized version in 2010) [119, 120]—achieved a better coverage of the human metabolic network, especially in compartments other than the cytosol. Recon 1 contains approximately 1.4 times more unique metabolites and 2.2 times more reactions than HumanCyc.

In 2013, a large improvement of the number of metabolic processes covered in the reconstruction was achieved with Recon 2 [121]. This is a consensus model that includes all reactions from EHMN, Recon 1, HepatoNet1 [122], and a module for acylcarnitine and fatty-acid oxidation [123]. Compared to Recon 1, Recon 2 represents a major improvement with approximately 1.7 times more unique metabolites and 2 times more reactions. Recon 2 was then refined in 2015 with updated gene-reaction associations. Simultaneously, a larger reconstruction named HMR (Human Metabolic Reaction database) [73] was built independently, based on HepatoNet1, Recon1, EHMN, Reactome, HumanCyc, KEGG [31], and the Human Metabolic Atlas [124]. HMR was then extended in 2014 to include lipid metabolism, therefore generating HMR2 [125].

Although containing a large set of reactions and being a consensus model, the predictions from Recon 2 were incorrect in some cases, mainly due to a number of incorrectly balanced reactions. At this point, the main focus of the community shifted to curation of existing models. Between 2014 and 2015, a series of minor updates and corrections were published for Recon 2 [126–129]. In 2016, Swainston et al. performed an extensive curation effort on Recon 2 and its updates, resulting in Recon 2.2 [130]. For the first time, a significant improvement of an existing model contained fewer genes than the original. In fact, due to the removal of duplicates and pseudogenes, and to the new unique HGNC ID convention, Recon 2.2 contains 112 enzyme-encoding genes less than Recon 2, and for the first time with unified identifiers (grouping splice isoforms into a single gene). Recon 2.2 is a major improvement for the energy-related metabolism, with a new compartment simulating the mitochondrial intramembrane space. As a result, the ATP and biomass flux predictions were greatly improved, achieving for the first time accurate predictions of maximum ATP yield under 14 carbon sources, both in aerobic and anaerobic conditions, and under 20 additional amino acid carbon sources in aerobic conditions [130].

In 2017, a new human metabolic model named iHsa [131] was obtained as an expansion and manual curation of HMR2. iHsa is a result of a reconciliation with the rat metabolic model iRno, also presented in the same paper. Both models were generated in parallel; rat-specific reactions, thermodynamically infeasible reaction loops, and other incorrect reactions present in HMR2 and Recon 2 were removed from iHsa, while new reactions were added from KEGG and MetaCyc/BioCyc [132]. For the first time, rat-specific reactions were removed from a human metabolic reconstruction. In fact, both models were reconciled and manually curated focusing on species-specific metabolic differences. The clear definition of differences between the two metabolic networks, coupled with the presence of unique reactions in both models, opens opportunities for human/rat cross-species comparison.

In 2018, Recon 3D [133] was developed from Recon 2 by incorporating HMR2 and a number of additional reaction sets, including reactions modelling host-microbe interaction, reactions for simulating drug effects on human metabolism, reactions for absorption of dietary compounds, reactions of lipid metabolism and reactions from metabolomics datasets. 3D protein structures, pharmacogenomics data and atom-atom mappings were also included in the model. After extensive manual curation steps and refinement of GPR rules, the model was tested for consistency when replicating 431 essential functions of the human body. To date, Recon 3D is the largest metabolic reconstruction available for human metabolism, containing 1.1 times more unique metabolites and 1.4 times more reactions than Recon 2.

**Figure 3:**
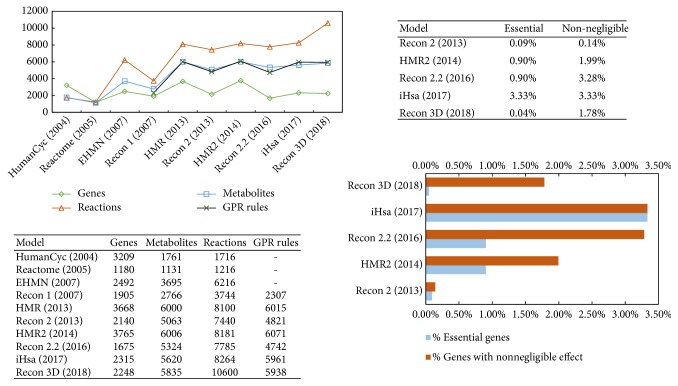
*(Top, left) Number of genes, metabolites, reactions, and GPR rules in human metabolic models.* The number of genes in the table refers to the number of transcripts in the model. (Number of enzyme-encoding genes: Recon 1: 1490, Recon 2: 1789, and Recon 2.2: 1675.) Recon 2 was considered in its latest Recon 2.04 iteration. The GPR rules in HMR have been extracted as all the nonempty “listOfModifiers” fields via a Matlab custom script. The boundary metabolites were excluded from the comparison, therefore metabolite counts represent the size of the stoichiometric matrix on which the model is built.* (Bottom, right) Gene essentiality in the five most recent metabolic models*. A gene is deemed essential if, when knocked out, it induces a biomass value lower than 10^−10^  h^−1^. A gene is nonnegligible if, when knocked out, it affects the biomass by more than 10^−10^  h^−1^. Although the improvement in the number of genes and metabolites has been limited in the past five years, the curation and gap filling efforts have produced more reliable models. As a result, compared to Recon 2, there has been a sharp increase in the percentage of genes whose knockout yields measurable effects on the predicted biomass.

Figure 3 summarizes the progress made by the metabolic modelling community over the last 15 years. The number of genes, metabolites and reactions is reported in the figure. To better quantify the connectedness of a model, we computed the number of genes whose knockout yields measurable effects in the model as measured by the predicted biomass flux. To account for the tolerance of the linear solver, we defined a gene* essential* if its knockout produces biomass <10^−10^  h^−1^; we defined a gene* nonnegligible* if its knockout causes a biomass variation >10^−10^ h^−1^. Compared to Recon 2, the increase in the number of genes whose variation has a nonnegligible effect on the growth rate shows that efforts have been successfully made towards curation, gap filling, and consistency checks, as detailed in Section 3.1.

### 3.1. From Size-Focused “Modelathons” to Manual Curation

When the first Recon 1 [118] and EHMN [119] human metabolic reconstructions were published in 2007, Recon 1 was preferred in many studies because it contained more reactions. In fact, this was only due to the compartmentalization of Recon 1, with metabolites repeated in different compartments and transport reactions between compartments. The number of unique reactions was indeed greater in EHMN (1028 more reactions and 1202 more metabolites), and a subsequent compartmentalized version of EHMN was generated [120].

These frequent comparisons among different models indicate that the main focus was initially to include as many reactions as possible—a size-focused “modelathon” approach—often at the expenses of curation and accuracy. Conversely, the approach of including all the available biological components in a model is now always coupled with extensive team-based curation, consistency checks, uniformity of annotations and gap filling. It is not uncommon to achieve a better model by removing reactions from an existing one. This encouraged the community to focus on the accuracy of the models and solvers used, rather than only on the model size.

Manual curation is therefore considered one of the main steps in generating a metabolic model. Tools like MetaNetX [134] simplify curation efforts and suggest identifier mappings to unify reactions and metabolites identifiers. Such curation efforts are likely to become even more important in the near future given the recent advances of automated reconstruction tools, now able to generate a full working draft of a model, e.g., MicrobesFlux [135], Pathway Tools [136], PathwayBooster [137], CoReCo [138], and Merlin [139]. Given the importance of obtaining reconciled and high-quality models, MetaNetX also provides a database of models generated after reconciliation of metabolites and biochemical reactions [134]. More recently, CarveMe [140] and RAVEN 2.0 [59] have shown high potential in automating manual steps, achieving for the first time an accuracy directly comparable with manually curated models when predicting experimental phenotypes. Tools for checking inconsistencies and for visually inspecting the model are also available [141].

Over the last few years, the curation efforts have yielded a reduction in the number of unbalanced reactions, as well as in the number of blocked reactions and dead-end metabolites. For instance, Recon 2.2 has no unbalanced reactions apart from the biomass objective functions, and Recon 3 has now less than 15 blocked reactions and dead-ends. Furthermore, as generating context-specific models using omics relies on GPR rules, the accuracy of these models strictly depends on the accuracy of the GPR in the baseline model. As shown in Figure 3, GPR coverage is increasing. Finally, the agreement between theoretical and model-predicted ATP yields has been improved in the most recent models. We calculated the root mean squared error (RMSE) between the theoretical and model-predicted maximum ATP yields per unit of carbon source, which has been reduced from *∞* (Recon 2 and earlier models) to 5.17 (in Recon 3) and 1.67 (in Recon 2.2). The table of ATP yields and the RMSE calculations are provided as Supplementary Material.

## 4. Tissue- and Patient-Specific Insights into Human Disease Metabolism

Starting from human generic metabolic reconstructions, a number of successful efforts have been published on reconstructing tissue-specific models. These include brain [142], adipocytes [143], breast cancer [144], heart [145], kidney [146], myocytes [147], and hepatocytes [125, 148]. A further set of 32 tissue- and organ-specific models has been generated by mapping protein expression onto the generic human model HMR2 [125].

Tissue-specific models are usually built as a reduction of a generic human model. The reactions that are removed in the construction of the tissue-specific model are found to be not active in that tissue. Reactions are removed according to transcriptomic or proteomic data collected in that tissue, and therefore the tissue-specific model contains fewer reactions than the generic one. Unsurprisingly, in most cases they outperform the generic counterpart where they stemmed from. At this point, a new tissue-specific biomass equation needs to be established, or the model needs to be set so that the cell can find alternative pathways to sustain its life. Although many methods for generating tissue-specific models set the goal of creating the minimal metabolic model that satisfies viability or a set of metabolic tasks, the use of nonminimalistic methods for generating tissue-specific models should be preferred. For instance, considering essentiality before removing reactions from a generic model yields a nonminimal tissue-specific model, but improves model functionality and agreement with experimental data [149]. Since the generic models are a superset of the tissue-specific models, predictions of generic models may still be correct in tissue-specific problems, but less accurate.

Context-specific human metabolic models have shed light on disease onset and progression in a range of recent case studies. In this context, the renewed interest in disease metabolism is due to the fact that a holistic genome-scale view, rather than a single-gene approach, is necessary to fully characterize most diseases. For instance, in cancer, the differences in the metabolic pathways between cancer cells and their parent tissue have been characterized using omics data and a human metabolic model [75, 150, 151]. As a result, tissue- and cell-specific metabolic models have been successfully used to identify—and successively validate—specific drug targets that inhibit cancer proliferation but do not affect normal cell proliferation [74, 152]. With a similar approach, submodels built from human genome-scale models have been used to generate several testable hypotheses, e.g., to compare wild-type and Fh1-deficient kidney mouse cells, and to predict further gene knockouts that affect growth in the Fh1-deficient cells but do not affect the wild-type cells, therefore suggesting targets for treating hereditary leiomyomatosis and renal-cell cancer [153].

For specific diseases, tissue-specific models have been used successfully to identify biomarkers and therapeutic targets [125]. Using RNA-Seq expression levels in combination with genome-scale models has enabled the reconstruction of cancer cell line-specific models, enabling the discovery of metabolites supporting proliferation, and antimetabolites leading to cell death [154]. (An antimetabolite is a compound that simultaneously inhibits those enzymes involved in metabolizing the associated endogenous metabolite. Antimetabolites can affect multiple enzymes at the same time and can reduce proliferation, and are therefore used as anticancer drugs.) After integration with extracellular metabolomic data and transcriptomic profiles, metabolic modelling has reliably characterized intracellular metabolism of lymphoblastic leukemia cell lines [155]. A combination of different omics data and flux splits have been used to generate cancer-specific models of the NCI60 panel, achieving correlation close to 1 in predicting the remaining flux rates [13].

Following the same direction, personalized models (e.g., using patients' omic data to constrain the generic reconstruction) hold promise to become key for precision medicine (Figure 2). The largest study to date with patient-specific models is the Human Pathology Atlas [77]. The authors built personalized genome-scale models of cancer in each patient across 17 tissues. This allowed investigating the metabolic differences between different cancers, as well as patient-specific biological functions. In another recent study [156], 86 patients with nonalcoholic fatty liver disease were recruited, and their personalized hepatocyte genome-scale metabolic models were built using patient-specific experimental data on lipoprotein fluxes. A new molecular mechanism of the disease was elucidated as a result of personalized metabolic modelling.

Genotyping coupled with patient-specific metabolic modelling offers a new opportunity for personalized medicine. In fact, new biomarkers can be predicted in a patient-specific fashion, and personalized therapies can therefore be designed and subsequently assessed in terms of their metabolic mechanistic effects [157]. As shown in a recent case of arginase deficiency (a urea cycle disorder), different individuals can respond differently to the same disease and treatment, and this can often be flagged observing their individual metabolic response [158].

Due to the importance of the gut microbiome composition in human health, a set of modelling approaches has been proposed recently [159]. Using host-microbiome modelling, e.g., combining metabolic community modelling with human metabolic models, the interplay among the gut bacteria and their interaction with the surrounding human cells can be investigated [160, 161]. Personalized models of gut microbiome have been built from patient-specific metagenomic data in order to elucidate individual-specific bile acid production in microbiomes of healthy individuals and patients with inflammatory bowel disease [162]. Although several modelling challenges remain to be solved [163], an approach based on metabolic modelling is likely to shed light on the role of human gut microbial communities in human health, therefore suggesting potential dietary changes or personalized intervention on gut composition [164, 165].

## 5. Discussion and Perspective

Research in computational biology has led to detailed models for a better understanding of the individual biological components, but arguably to a less clear picture of the interactions among the components that result in a given phenotype [166]. Genome-scale systems biology studies can effectively address this issue. For instance, in biomedical applications, this holistic view is necessary to characterize a patient's disease phenotype and deliver precision medicine [17].

Since metabolic homeostasis and observable phenotype are strictly linked, metabolism is nowadays considered diagnostic of the phenotype, and therefore arguably the best indicator of the functional state of a cell. Metabolism can also be used to prioritize genes and assess their function and the role of gene perturbations (including knockouts). Without such integrated analysis, a gene may e.g., incorrectly be regarded as important only due to its highly variable expression value.

Metabolic models are increasingly being used to construct multiscale, multicellular or multitissue models. In 2012, a research effort by Karr et al. [21] provided the first whole-cell computational model of the life cycle of a small pathogenic bacterium,* Mycoplasma genitalium*. The model includes metabolism, replication of the genome, and cell division. Several metabolic models can be combined in frameworks to investigate the metabolic exchanges between individual cells and the emerging community behavior [167], with applications ranging from microbial communities to host-pathogen interactions and cancer proliferation [168–170].

The availability of tissue-specific models has also led to the generation of multitissue metabolic models [171]. Further steps in this direction will soon allow studying whole-body metabolism models. The first study towards this goal used dynamic parsimonious FBA to combine steady-state FBA with differential equations for the concentration of metabolites in each organ [172]. As a result, a whole-body and inherently multiscale model was generated with 14 organs plus the human serum. More recently, the first whole-body metabolic models were generated named Harvey and Harvetta, where the metabolic network for every organ/tissue was built simultaneously from Recon3D [173]. Manufacturing organ-on-a-chip and ultimately body-on-a-chip devices with metabolic models (see e.g., [174]) seems the next step in this direction.

Given the recently renewed interest of the scientific community in understanding disease metabolism, it is highly likely that cancer metabolism (and metabolism in other diseases) will become the main research topic in drug development. This will complement rather than replace standard transcriptomic-only studies, and will provide a proxy for the phenotypic and observable outcome. The idea is that to improve phenotypic predictions, the signatures need to be taken from sources of data that are closer to the phenotype. The integration of omics data into metabolic models has enabled the prediction and successively the validation of biomarkers and therapeutic targets. In drug design, although genomics, transcriptomics and proteomics are often deemed sufficient, metabolomics can elucidate mechanisms that are not visible from genes and protein activity. More importantly, it can address cases where the genes responsible for a disease are well known but not druggable, but their corresponding downstream reactions are [175].

Genomics analysis is usually able to identify known diseases through gene mutations. However, it may not be able to flag variants of known diseases, or identify novel diseases. This can happen, for instance, if (i) a gene mutation is not flagged as important; (ii) the gene is not screened at all; (iii) the disease is not a direct effect of a gene perturbation; (iv) individuals respond differently to the same mutation [158]. Genome-scale models can elucidate mechanistic modes of drug action, side effects (both off-target drug binding and downstream transcriptional effects), and potential toxicity of drugs by linking omics data to the phenotype through a condition-specific model [8, 176]. In a patient-specific framework, such biological data and markers can be predicted in a personalized fashion, paving the way for* in silico* clinical trials [177].

The efforts of the metabolic modelling community are far from being complete. In this regard, we envisage a joint effort from metabolic modelling and genome-wide association studies (GWAS) communities to identify gene-metabolite interactions that are currently not included in metabolic models using state-of-the-art association mappings [178]. Computational tools combining both approaches would go towards genome-scale detection of errors and missing enzymatic reactions, remarkably improving the predictive ability of metabolic models. For instance, this could be achieved by minimizing the error between predictions of flux coupling and experimental coexpression data integrated with GWAS [179, 180].

Likewise, the methods for integration of omics data in genome-scale models still show room for improvement. For instance, in yeast and* Escherichia coli*, single-omic integration in FBA has been reported to give similar accuracy to pFBA without omic integration [78]. Reassuringly, reaction-specific rules to constrain flux rates can halve the normalized error of pFBA [84]. In multicellular organisms, a better correlation between gene expression and metabolic fluxes can be expected [78]. In mammalian cells, the main contributors to the overall protein expression level are mRNA levels [181, 182], while in most normal and cancer cell lines, mRNA and protein levels were found to correlate positively [183, 184]. While different methods (and their parameters) can yield different context-specific models, the accuracy in predicting essential genes is almost always higher in context-specific models than in the generic human models [81]. Therefore, metabolic models can be regarded as useful tools to mechanistically link transcriptomic data with flux rates. Using multiple omics data and thermodynamic constraints simultaneously [185, 186], or a combination of regularized FBA methods and omics data, can improve the reliability of the predictions [187].

Compared to the well-characterized microbial metabolic reconstructions, gaps still present in our knowledge of human metabolism—including characterization of enzymes, the definition of cell-specific metabolic functions and tissue-specific growth mechanisms—make it more difficult to test (and compare) metabolic models and omic integration methods. Human metabolic models and methods have been tested and cross-compared for gene essentiality through CRISPR-Cas9-mediated loss-of-function screens, and for their ability to predict growth rate and recapitulate known metabolic functions [81]. A dataset including both omics data and measured metabolic information is the NCI-60 panel, with metabolite uptake and secretion rates published in 2012 [188]. While* E. coli* and yeast fluxomic data is publicly available from several experiments [189–191], to the best of our knowledge NCI-60 is the only publicly available human-cell dataset containing expression levels, metabolic flux rates and proliferation rates, and therefore suitable for validating FBA methods. Due to the lack of fluxomic datasets, the difficulty in choosing a reliable objective function, and the larger size compared to bacterial models, further experimental validation is needed for human metabolic models, especially when using the full flux distribution to inform decisions or subsequent algorithmic steps.

Finally, despite many recent advances, gathering insights from the data generated through omic-informed models remains a bottleneck in systems biology. We therefore envisage that genome-scale metabolic models will be increasingly investigated with machine/deep learning algorithms in a patient-specific fashion [13, 192]. In disease modelling, the usefulness of general biomarker discovery is debatable when information on patients is not taken into account, because most biological components would be flagged as perturbed in a general “disease versus normal” analysis. Conversely, if the appropriate person-specific data is integrated with a model, biomarkers can become the central part of precision medicine, where data acquired on patients drive predictions, analysis and therapeutics [17]. In a modelling analogy, machine learning algorithms alone cannot provide mechanistic information in the biological processes they simulate or mimic. However, if machine learning is coupled with multiomic genome-scale modelling, the combination of experimentally and model-generated omic data can predict—and explain mechanistically—personalized therapy predictions by including key biological information in the learning process.

## Supplementary Materials

Supplementary Materials Agreement between theoretical and model-predicted ATP yields for the most recent human metabolic models. We calculate the root mean squared error (RMSE) between the theoretical and model-predicted maximum ATP yields (per unit of carbon source) for the most recent human metabolic models. The RMSE has improved from ∞ (Recon 2 and earlier models) to 5.17 (in Recon 3) and 1.67 (in Recon 2.2).
